# The effect of smartphone addiction on adolescent health: the moderating effect of leisure physical activities

**DOI:** 10.1186/s41155-024-00308-z

**Published:** 2024-06-27

**Authors:** Rong Zhang, Qimeng Jiang, Meichao Cheng, Yong-Taek Rhim

**Affiliations:** 1 Department of Sport and Leisure Studies, Namseoul University, Cheonan, Chungcheongnam-do Korea; 2 University of Glasgow, Glasgow, UK; 3 School of Physical Education, Shandong University, Jinan, China

**Keywords:** Smartphone addiction, Leisure physical activities, Adolescents, Health constitution

## Abstract

**Background:**

Smartphone addiction is a growing concern, especially among adolescents, due to its negative impact on health. This study examines how leisure physical activity influence this relationship.

**Objective:**

This study aimed to investigate the relationship between smartphone addiction and adolescent health, examining the mediating role of loneliness and the moderating effect of leisure physical activity.

**Methods:**

A survey of 1271 Chinese university students was conducted using the PAPS Health and Fitness Scale, Smartphone Addiction Scale, and Leisure Time Exercise Scale.

**Results:**

Smartphone addiction negatively affects adolescent health. Loneliness mediates this relationship, while leisure physical activity moderates it. High levels of physical activity reduce the negative impact of loneliness on health (bsimple = −0.49, *P* < 0.001), whereas low levels enhance this impact (bsimple = −0.21, *P* < 0.001).

**Conclusion:**

These research have practical implications for preventing and reducing smartphone addiction and offer a theoretical foundation for promoting healthier lifestyles among adolescents.

## Introduction

Smartphone addiction, characterized by a persistent and intense dependence on mobile phones for interpersonal interaction, has become increasingly prevalent with the widespread adoption of smartphones (Moccia et al., 2017). This phenomenon has raised significant concerns due to its association with various adverse effects, including sleep deprivation and negative impacts on both physical and mental health (Aktürk et al., 2018). A research report (Liu, 2023) highlighted the alarming prevalence of smartphone addiction among students in China, linking it to lower academic performance, physical inactivity, poor sleep quality, and increased risk of overweight/obesity, as well as musculoskeletal issues such as shoulder, eye, and neck pain (39.2%, 62.2%, and 67.7%, respectively) and serious mental health disorders (30.7%). Notably, the report revealed variations in smartphone addiction rates across different regions, with higher prevalence observed in rural areas compared to urban and town areas (17%, 12%, and 11%, respectively). The consequences of smartphone addiction extend beyond physical health issues, including vision problems and bone deformation (Makenzie et al., 2018), to encompass significant mental health challenges such as depression and anxiety, which can adversely affect interpersonal relationships (Li et al., 2022). While some scholars argue that “smartphone addiction” may not constitute a genuine addiction but rather reflects a fundamental human desire for social connection (Veissière & Stendel, 2018), the detrimental effects associated with excessive smartphone use are well-documented.

Overreliance on smartphones during adolescence can bring a variety of problems which, on the one hand, will do harm to physical health such as low vision and bone deformation; on the other hand, mental problems such as depression and anxiety will affect interpersonal relationships (Darcin et al., 2016). Studies also found that excessive use of smartphones by university students had negative academic, psychological, and physical impacts (Zhang et al., 2014). Individuals addicted to smartphones show psychological symptoms such as compulsive behavior, tolerance, withdrawal, and anxiety (Nathan & Zeitzer, 2013). Feldman believed that loneliness was an emotion caused by unsatisfied social motivation and gregarious behavior (Feldman et al., 2016) and is often accompanied by loneliness, helplessness, depression, and a sense of emptiness (Grosclaude & Jean-Luc, 2010). Among college students, many people have varying degrees of loneliness (Agnew, 1992). Mood regulation (defined as avoiding/reducing negative feelings—loneliness, anxiety, depression, stress) had a significant positive effect on smartphone addiction among a convenient sample of 394 Chinese university students (Cui et al., 2021). Likewise, loneliness score showed significant positive correlation with smartphone addiction score and emerged as an independent predictor of cyberspace-oriented relationship score, in a sample of 367 Turkish university students (Fantinelli et al., 2023). Literature suggests that overusing social media, instant messaging, and e-mail communication instead of in-person interactions are more likely to lead to social isolation triggering stress. Loneliness is more common among modern students than ever (Ikeda and Nakamura, 2014; Kim et al., 2017). At the same time, some studies on exercise psychology have also found that physical activity is an effective way to improve physical and mental health (Lei et al., 2020). Short-term appropriate physical activity can reduce negative emotion and induce positive emotion (Janssen & LeBlanc, 2010), while long-term appropriate physical exercise can alleviate loneliness in adolescents (Aljomaa et al., 2016). A consistent relationship has been demonstrated between SA and physical and mental health symptoms (Durak, 2017), including depression, anxiety, musculoskeletal problems (Kuss & Griffiths, 2017), and poor sleep (Muhammad et al., 2020). It is of great significance for maintaining the physical and mental health of adolescents to explore the mechanism of smartphone addiction among adolescents (Gallardo et al., 2023), thereby reducing the level of smartphone dependence, increasing the leisure sports activities, and improving physical fitness.

**Fig. 1:**
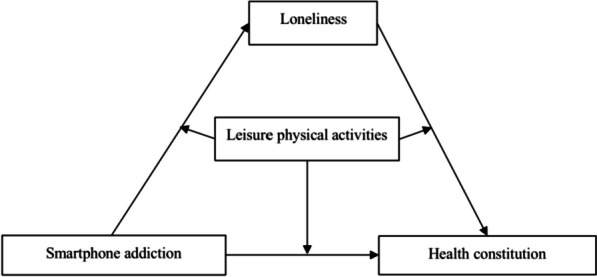
Research theoretical models

This study aims to uncover the mechanisms by which smartphone addiction affects loneliness among adolescents and explore the potential for intervention through leisure physical activities. The findings will enhance the understanding of the sociopsychological impacts of smartphone addiction, particularly on adolescent mental health. The results provide empirical evidence for interventions and policy development. For instance, schools and communities can promote leisure physical activities to help adolescents reduce smartphone usage, thereby improving mental health. Additionally, policymakers can use these insights to formulate regulations that manage smartphone use and mitigate its negative effects. Based on the analysis, this study proposes a moderated mediation model (as shown in Fig. 1).

## Method

### Participants

From the initial convenience sampling, including students from 8 universities in the Zhejiang province of China, 1.271 valid protocols (836 males and 435 females) were selected by stratified random cluster sampling, and the questionnaires were filled as self-report.

### Instruments

#### Survey of sociodemographic characteristics

A questionnaire was designed that included various sociodemographic elements and biographical data of the participants, such as age, sex, grades, and household income (10,000 yuan).

#### Leisure time exercise scale

The leisure physical activity of college students was assessed by the Godin Leisure Time Exercise Scale (Godin & Shephard, 1985; Renninger et al., 2023; Nathan, 2013). The scale consists of four items, with the first three questions designed to investigate how many times the college students participated in at least 15-min mild, moderate, and strenuous leisure physical activities in a week. The total number of leisure physical activities in 1 week was calculated by the formula, which was expressed as follows: exercise index = (frequency of light exercise × 3) + (frequency of moderate exercise × 5) + (frequency of vigorous exercise × 9), and the participants with the score of lower than 24 would be considered to have insufficient participation in leisure sports activities. The results of confirmatory factor analysis showed good fitness with fitness indices of Q: 2.156, GFI: 0.928, CFI: 0.961, RMR: 0.047, and NFI: 0.973, and the Cronbach’s alpha coefficient of the scale was 0.90, indicating that the questionnaire has good structural validity.

### Social loneliness scale

UCLA Loneliness Scale (UCLA Loneliness Scale (version 3)). The third edition of the UCLA Loneliness Scale edited by Russell et al. in 1996 was used to evaluate the loneliness caused by the gap between the desire for social interaction and reality (Russell, 1996; Yan et al., 2023; Xie, 2019). The Cronbach alpha coefficient was 0.89, and the correlation coefficient was 0.73. The results of confirmatory factor analysis (CFA) showed that the questionnaire had good construct validity: (2/df = 13.47, *NFI* = 0.98, *CFI* = 0.94, *GFI* = 0.99, *RMSEA* = 0.06), which has 20 items, including 11 “loneliness” positive-order items and 9 items of “non-loneliness” in reverse order. The scale used a 4-point score, in which the higher score suggested the stronger level of loneliness.

#### Smartphone addiction scale

In this study, the Mobile Phone Addiction Index (MPAI) (Kwon et al., 2013), compiled by Lei and translated into Chinese by Lu (Lei et al., 2020; Lu et al., 2021), was employed for assessment. The self-diagnosis scale categorizes individuals based on their total scores: those scoring 44 or above are classified as a high-risk user group, scores between 40 and 43 are considered a potentially dangerous user group, and scores below 39 are categorized as an ordinary social-content group. To analyze the relationship between different scores, the 1271 collected data points were divided into four equal parts, as illustrated in Table 1. The reliability of the data obtained in this study, measured using Cronbach’s alpha, was 0.86.

**Table 1:** Gender, annual household income, physical fitness, and level of smartphone use

	**Total**	**Q1 (≤ 23)**	**Q2 (24~29)**	**Q3 (30~34)**	**Q4 (35 ≤)**	***p*-value**
No. (%)	1271	346 (27.1%)	341 (26.8%)	310 (24.4%)	274 (21.6%)	
Sex						< 0.001
Male	836 (65.8%)	250 (30.0%)	232 (27.8%)	206 (24.6%)	148 (17.7%)	
Female	435 (34.2%)	84 (19.4%)	104 (23.9%)	104 (23.9%)	143 (32.9%)	
Grades						0.207
Freshman	295 (23.2%)	83 (28.1%)	55 (18.6%)	79 (26.7%)	78 (26.4%)	
Sophomore	339 (26.7%)	71 (20.9%)	99 (29.2%)	73 (21.5%)	96 (28.1%)	
Junior	316 (24.9%)	74 (23.4%)	89 (28.2%)	82 (25.9%)	48 (15.2%)	
Senior	321 (25.3%)	98 (30.5%)	98 (30.5%)	76 (23.7%)	52 (16.2%)	
Household income (10,000 yuan)						0.652
*n* ≤ 10	266 (20.9%)	66 (24.8%)	78 (29.3%)	72 (27.1%)	50 (18.8%)	
10 < n ≤ 20	699 (55.0%)	218 (31.2%)	171 (24.5%)	167 (23.4%)	143 (20.5%)	
20 < n ≤ 40	180 (14.2%)	45 (25.0%)	49 (27.2%)	44 (24.4%)	42 (23.3%)	
40 < n	126 (9.9%)	39 (31.0%)	38 (30.2%)	17 (13.5%)	32 (25.4%)	
Health constitution (point)						0.100
≤ 63	637 (50.1%)	160 (25.1%)	170 (26.7%)	157 (24.6%)	150 (23.5%)	
64 ≤	634 (49.9%)	187 (29.5%)	171 (27.0%)	154 (24.3%)	122 (19.2%)	

#### PAPS health physique

The “PAPS Health Physique” assessment is based on the Physical Activity Promotion System (PAPS) as detailed by He (He et al., 2020). This comprehensive evaluation tool is designed to measure students’ health and physical fitness across five key physique factors: cardiopulmonary endurance, flexibility, muscular endurance, explosive force, and BMI. Cardiopulmonary endurance is assessed through lung capacity measurements, flexibility through seated forward bending tests, muscular endurance through an 800-m run, and explosive strength through a 50-m sprint. Each factor is scored on a scale from 0 to 20 points, with the total possible score being 100 points. The validity and reliability of the PAPS have been confirmed with a CFI of 0.905, RMSEA of 0.053 (0.047, 0.058), and a Cronbach’s alpha of 0.90, indicating a high level of internal consistency and measurement accuracy.

### Statistical analysis plan

The data in this study were processed using the SPSS 25.0 and Amos 24.0 statistical software. Firstly, the valid data were imported into SPSS 25.0 analysis software. To examine the relationships between social demographic variables, a cross-tabulation analysis was conducted, and differences were tested using the chi-square test, and the K-S nonparametric test, reliability analysis, and exploratory and validation factor analysis were used to test the normal distribution of the data and the reliability and validity of the instruments. Amos 24.0 was used to validate the scale’s structural validity. To further investigate the degree of smartphone dependence and varying levels of health constitution, logistic regression analysis was employed. This analysis adjusted for potential confounders, including gender and household income. A *p*-value of less than 0.05 was considered statistically significant. The odds ratios (OR) were reported with 95% confidence intervals (CI), and the significance level (α) was set at 0.05. And mediated effects model was fitted. Correlation analysis, regression analysis method, and the macro program PROCESS plug-in in SPSS 25.0 were used for the chained mediation effect test and bootstrap analysis test. The significance level of the statistical results was set at *P* < 0.05.

## Results

### Descriptive statistical analysis

First, descriptive statistical analysis on every variable was carried out to examine whether there was statistical significance in gender, annual household income, and health constitution (as shown in Table 2).

**Table 2:** The link between levels of smartphone addiction and health constitution

**Smartphone addiction levels**	**Health constitution (≤ 63)**	
	**Crude model**	**Multi-adjusted model**
	**OR (95% *CI*)**	**OR (95% *CI*)**
Q1 (≤ 23)	1.00 (ref.)	1.00 (ref.)
Q2 (24～29)	1.15 (0.87～1.53)	1.21 (0.93～1.61)
Q3 (30～34)	1.21 (0.90～1.61)	1.25 (0.93～1.65)
Q4 (35 ≤)	1.47 (1.07～1.89)*	1.62 (1.18～2.15)*

Multi-adjusted model: sex and household income

*P<0.05, **P<0.01, ***P<0.001 (similarly hereinafter)

### Common method *bias* test

To minimize common method bias inherent in self-report data collection, this study employed anonymity and reverse scoring for some items. After data collection, the Harman single-factor test was conducted to assess common method bias (Wei et al., 2023). The results revealed 18 factors with eigenvalues greater than 1, and the first factor explained 28.21% of the variance, which is below the 40% threshold, indicating no serious common method bias in the study. We found a significant negative correlation between smartphone addiction and health constitution (*r* = −0.32, *P* < 0.001). After adjusting for demographic variables such as age, gender, and household income, the relationship remained significant (adjusted *r* = −0.28, *P* < 0.001). These results underscore the robust negative impact of smartphone addiction on health constitution, independent of demographic factors.

### The relationship between healthy constitution and smartphone addiction

The relationship between health physique and smartphone addiction is shown in Table 2 when the smartphone addiction score was 24–29 points. For instance, we describe the odds ratio (OR) as a measure indicating the odds of an outcome occurring with a one-unit increase in the predictor variable and confidence intervals (CI) as a range within which the true effect size is expected to fall with 95% confidence. The probability of health physique score lower than 63 points was 1.15 times higher (95% *CI*: 0.87–1.53). When the smartphone addiction score was over 35, the odds of a health physique score lower than 63 points were 1.47 times higher (95% *CI*: 1.07–1.89), with a statistical difference (*P* < 0.05). In addition, after adjusting gender and annual household income and the smartphone addiction score was 24–29 points, the probability of a healthy physique score lower than 63 was 1.21 time higher (95% *CI*: 0.93–1.61), with no statistical difference. When it suggested 30–34 points, the probability of a healthy physique score lower than 63 points was 1.25 times higher (95% *CI*: 0.93–1.65), and there was no statistical difference. If the smartphone addiction score exceeded 35 points, the probability of health physique score lower than 63 points was 1.62 times higher (95% *CI*: 1.18–2.15) and with a statistical difference (*P* < 0.05).

### Descriptive statistics and correlation analysis of variables

The mean, standard deviation, and correlation coefficient of each variable are presented in Table 3. Research suggests that regular physical activity benefits physical, social, and mental health (Mahapatra, 2019). Smartphone addiction was significantly positively correlated with loneliness and significantly negatively correlated with leisure physical activity and a healthy physique. Loneliness was negatively correlated with leisure physical activity and significantly negatively correlated with a healthy physique. Leisure physical activity was significantly positively correlated with a healthy physique (*P* < 0.001).

**Table 3:** Mean, standard deviation, and correlation matrix for each variable (*n* = 1271)

	***M***	***SD***	**1**	**2**	**3**	**4**
1. Smartphone addiction	35.76	9.32	1			
2. Loneliness	4.89	1.06	0.18**	1		
3. Leisure physical activities	112.73	15.41	−0.13**	−0.09*	1	
4. Health constitution	62.55	11.31	−0.29***	−0.20**	0.69***	1

*P<0.05, **P<0.01, ***P<0.001 (similarly hereinafter)

### Moderated mediation model test

In order to test the mediating effect of loneliness in smartphone addiction on adolescents’ health and to investigate whether the mediating effect of loneliness was mediated by leisure sports activities, the analysis was conducted according to the moderated mediation test proposed by Wen Zhonglin (Xie et al., 2019). After standardizing the data, gender and family income were used as control variables, and the data was analyzed in combination with the SPSS macro program PROCESS 3.3 used by Hayes (Ottosen et al., 2023). The results are shown in Table 4, and the effect was significant (*β* = −0.13, *t* = −5.38, *P* < 0.01), indicating that the more severely smartphone dependence was, the worse the health became. The results of Eq. 2 showed that the addiction of smartphones had a significant predictive effect on loneliness (*β* = 0.43, *t* = 13.65, *P* < 0.001), indicating that the more severely the mobile phone dependence of adolescents was, the stronger their loneliness could be. In Eq. 3, the effect of loneliness on health constitution was significant (*β* = 0.21, *t* = 18.21, *P* < 0.01), indicating that the stronger the loneliness of adolescents became, the worse the health constitutions were.

**Table 4:** Moderated mediation test

	Equation 1 (effective standard: health constitution)		Equation 2 (effective standard: loneliness)		Equation 3 (effective standard: health constitution)	
	***β***	***t***	***β***	***t***	***β***	***t***
Sex	−0.02	−0.46	0.01	0.12	−0.03	−0.47
Household income	0.26	2.43	0.19	0.45	0.07	2.35
Smartphone addiction	−0.13	−5.38**	0.43	13.65***	−0.02	3.05
Leisure physical activities			−0.28	−11.99***	0.33	2.19
Loneliness	0.21	−8.29***			0.21	18.21***
Smartphone addiction × leisure physical activities			−0.04	−0.27***	0.35	4.97*
Loneliness × leisure physical activities					0.06	3.82***
*R*^2^	0.11		0.37		0.24	
*F*	43.28***		421.68***		79.32***	

*P<0.05, **P<0.01, ***P<0.001 (similarly hereinafter)

Therefore, loneliness mediated the relationship between mobile phone addiction and physical fitness. In Eq. 3, the interaction term of smartphone dependence and leisure physical activity had statistical significance on health physique (*β* = 0.35, *t* = 4.97, *P* = 0.048). The interaction term of smartphone addiction and leisure physical activity had a significant predictive effect on loneliness (*β* = −0.04, *P* < 0.01), which indicated that leisure physical activity played a moderating role in the direct path of smartphone addiction on loneliness. The interaction item of loneliness had a significant positive prediction on the healthy constitution of adolescents (*β* = 0.06, *t* = 3.82, *P* < 0.01), indicating that leisure physical activity had a moderating effect on the relationship between loneliness and healthy constitution. In conclusion, the moderated mediation model of this study was established.

**Fig. 2:**
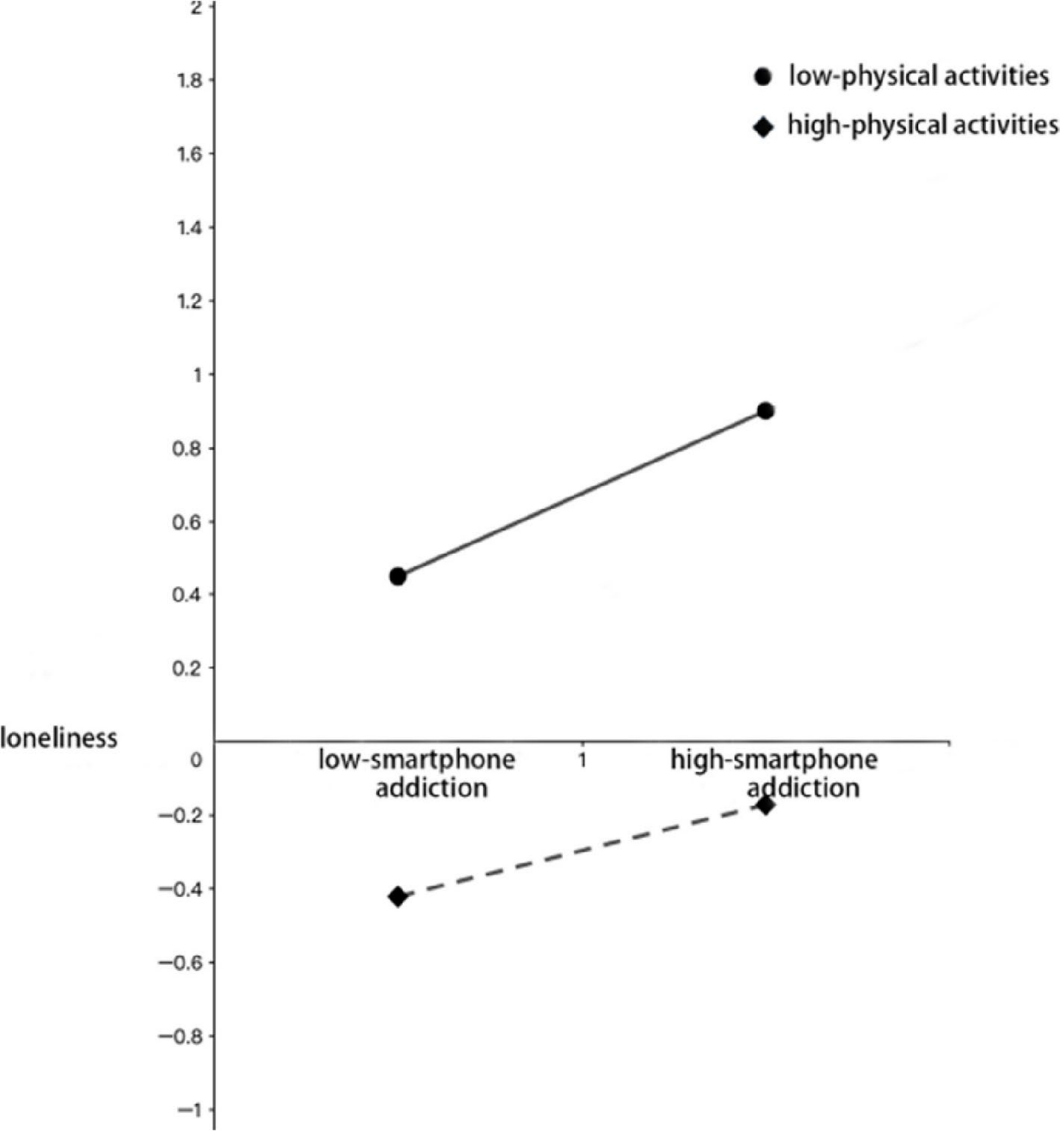
The moderating effect of leisure physical activity on the relationship between loneliness and smartphone addiction

In order to analyze the moderating effect of leisure physical activity, a simple slope test was used to examine the moderating effect of leisure physical activity on the relationship between loneliness and smartphone dependence (Fig.2).

**Fig. 3:**
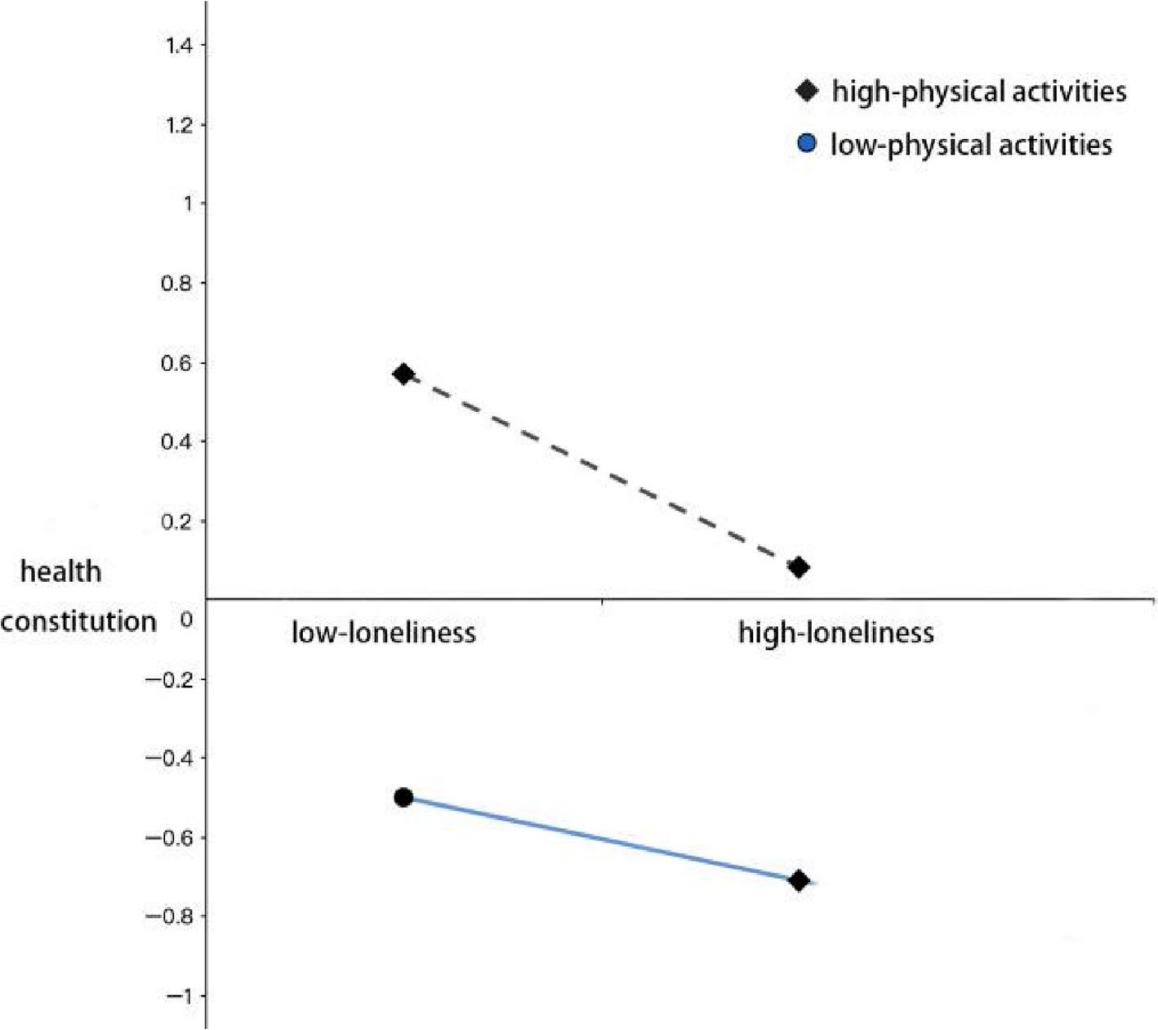
The moderating effect of leisure physical activity on the relationship between loneliness and healthy constitution

When the individual’s leisure physical activity level was low, smartphone dependence had a positive and significant predictive effect on loneliness, with a simple slope *β* = 0.43 (*t* = 5.49, *P* < 0.001); at the high level of leisure physical activity, although smartphone dependence remained its positive predictive effect on loneliness, the predictive effect was less, with the simple slope *β* 0.27 (*t* = 2.33, *P* < 0.01). Therefore, the influence of smartphone dependence on loneliness in adolescents varies according to the level of leisure physical activity. As a whole, with the increasing smartphone dependence level, the adolescents with high-level leisure physical activity felt less lonely than those with low-level leisure physical activity. Also, their healthy constitutions were better than those with low-level leisure physical activity. In order to explain more clearly the moderating effect of leisure physical activity in the second half, a simple slope analysis was conducted. As can be seen in Figure 3, when the level of leisure physical activity was high, loneliness had a significant negative predictive effect on physical fitness (bsimple = −0.49, *t* = −4.32, *P* < 0.001); the negative predictive effect of loneliness on physical fitness remained significant with the enhanced predictive effect (bsimple = −0.21, *t* = −12.66, *P* < 0.001). It showed that high-level leisure physical activity had a greater protective effect on health constitution in different states of loneliness, compared to low-level leisure physical activity.

## Discussion

### The influence of smartphones addiction on the healthy constitution

The results of this study show that adolescents’ smartphone dependence significantly predicts a decline in physical health, consistent with research indicating that comprehensive exercise-based interventions alleviate symptoms of smartphone reliance and improve sleep quality among university students (Nicola et al., 2017). Severe smartphone addiction is associated with lower health levels (Tang & Lee, 2021). This has been validated in adolescents, demonstrating that smartphone dependence can predict a decline in physical fitness across different age groups. Excessive smartphone use shares mechanisms with other addictive behaviors, such as gambling disorder, including reduced cognitive control and impaired prefrontal cortex activity, which affects decision-making and emotional processing (Sohn et al., 2021; Jafari et al., 2019). Adolescents with addictions tend to experience poor emotional regulation, impulsivity, impaired cognitive control, and reduced ability to derive pleasure from everyday life (Kumcagiz & Gunduz, 2016). Several studies have examined the negative effects of social network overuse, linking it to depression, difficulty in face-to-face communication, need for immediate rewards, neglect of offline relationships, professional problems, and loneliness (Benítez-Sillero et al., 2023). This study found no significant association between annual household income and smartphone dependence, which is inconsistent with González and Molero (2023). This discrepancy may be due to the study’s focus on university students in Zhejiang province, a limited sample size, and the exclusion of environmental factors beyond family income.

### The mediating effect of loneliness

Based on further verification of the relationship between smartphone addiction and adolescent health, this study explores the mediating role of loneliness in the relationship between the two. Meanwhile, this study has found that smartphone addiction has a significant effect in direct relation to physical health, and it can reduce the level of their physical health by promoting loneliness. Loneliness plays a mediating role in the effect of smartphone addiction on adolescent health. First, smartphone addiction positively predicted adolescents’ loneliness. There are very few social and sports facilities that allow students to interact socially on the university campus (Saud et al., 2022). The interaction theory model points out that the occurrence of psychological behavior is the result of the interaction between the individual and the environment (Sepúlveda et al., 2020). Investigations have found that university students are more likely to express themselves on the Internet, such as excessive immersion in virtual situations (Kim et al., 2015), which can lead to smartphone dependence. Especially for teenagers in university, they have seen the changes of learning environments through the transition from high school to university (Xiao et al., 2019). The rapid development of self-cognition makes it easier for teenagers at this stage to feel the gap between reality and ideal life so that they want to escape from the reality and feel lonely as well. This will lead to alternative satisfaction through playing smartphones and an increased risk of smartphone dependence (Gao et al., 2024). The personality characteristics of smartphone addicts prevent their basic needs from being satisfied in reality, which promotes the feeling of loneliness among university students in real life (Jiang & Zhao, 2016). It is known that the excessive use of smartphones has led to changes in lifestyles and differentiation of social interaction styles and also procured them to restrict their lives with the technological world (Sun et al., 2021). Individual communication skills and the real-world friendships of students affect adversely as a result of the use of social media networks and because students may choose their friends from the virtual environment instead of real life. Second, the level of loneliness can predict the physical health of adolescents (Thomée et al., 2011). This may be due to the fact that university students with high level of loneliness cannot handle their life pressure and negative emotions, resulting in their irrational perceptions, failure to share bitter and sweet in life, which may have an influence on young people’s positive cognition and experience of life (Mosalanedjad et al., 2019), and harm their physical health.

Therefore, we should pay more attention to the emotions of university students, increase relevant courses of health education, learn to regulate their emotions, and establish self-awareness of mental health, so as to prevent them from indulging in playing smartphones and becoming dependent on the Internet.

### The moderating effect of leisure physical activity

This study confirms that the level of leisure physical activity modulates the mediating pathway of “smartphone addiction → loneliness → physical health.” Leisure physical activity moderates the relationship between smartphone addiction and adolescent loneliness; for instance, individuals with lower levels of leisure physical activity are more likely to experience increased loneliness due to smartphone addiction. One reason is that adolescents with lower leisure physical activity are less adaptable to their social environment and may fear rejection or feel anxious about maintaining relationships, leading them to use smartphones to meet their security needs (Evgenia et al., 2024). Group counseling, combined with exercise, can suppress the psychological craving, addiction, and comorbid depression and anxiety of smartphone addicts to varying degrees (Coyne & Woodruff, 2023). Previous studies have suggested that self-awareness is a protective factor for various psychological variables. However, when self-awareness interacts with risk factors such as smartphone addiction, lower levels of leisure physical activity can exacerbate the perceived gap between expectations and reality, resulting in negative emotions such as loneliness and a greater tendency to seek psychological comfort and support through the Internet, thereby increasing smartphone dependence.

## Conclusions

This study examines the relationship between smartphone addiction and health constitution among adolescents, with a focus on the mediating effect of loneliness and the moderating effect of leisure physical activities. Our findings reveal significant insights into how physical activity can improve adolescent health through psychological mechanisms. We found that social isolation may lead to increased smartphone use as adolescents seek social connection, which in turn exacerbates feelings of loneliness and reduces physical activity. This vicious cycle negatively impacts health outcomes, illustrating the complex interplay between these factors. Our conclusions underscore the importance of further research into the dynamics between smartphone addiction and health. Future studies should explore additional mediators, such as self-esteem and social support, and moderators like gender and socioeconomic status. This deeper investigation will provide a more comprehensive understanding of the relationship between smartphone addiction and health. By focusing on adolescents addicted to smartphones, future research can expand on our findings to identify more mediating and moderating variables. This will help uncover various psychological mechanisms through which smartphone addiction affects adolescents, ultimately providing a theoretical basis for promoting healthier lifestyles among this population.

## Data Availability

The original contributions presented in the study are included in the article, and further inquiries can be directed to the corresponding authors. We can confirm that relevant research ethics committee guidelines for a project involving human participants were followed and approved by the Institution Review Board of the Department of Sport and Leisure Studies at Namseoul University of Korea. All participants gave informed consent.
